# Directed evolution of chlorotoxin enhances MMP-2 recognition and improves CAR-T-cell activity in glioblastoma models *in vitro*

**DOI:** 10.3389/fbioe.2026.1866928

**Published:** 2026-07-20

**Authors:** Anna Hajdara, Árpád Szöőr, József Murányi, Attila Brunyánszki, Gábor Z. Rácz, Daniel Cioca, Ambrus Gordos, Natali Bata, Noémi Nagy, Imre Fedorcsák, László Sipos, Máté Kisgyörgy, Márton Megyeri, Zalán Péterfi, Sándor Farkas, Péter Hornyák

**Affiliations:** 1 VRG Therapeutics Ltd., Budapest, Hungary; 2 Department of Biophysics and Cell Biology, Faculty If Medicine, University of Debrecen, Debrecen, Hungary; 3 Doctoral School of Biology, ELTE Eötvös Loránd University, Budapest, Hungary; 4 Department of Neurosurgery and Neurointervention, Semmelweis University, Budapest, Hungary

**Keywords:** CAR T-cell, chlorotoxin, glioblastoma, MMP-2, phage display, protein engineering

## Abstract

**Background:**

Cysteine-rich miniproteins from venomous animals are promising therapeutic scaffolds due to their compact structure, high stability, and low immunogenicity. Chlorotoxin (CTX), a miniprotein derived from scorpion venom, selectively targets glioblastoma by binding matrix metalloproteinase-2 (MMP-2), an invasion-associated enzyme overexpressed in ∼80% of glioblastomas. CTX has been utilized in tumor imaging, drug delivery, and immunotherapy, including its incorporation into chimeric antigen receptor (CAR) constructs. CTX-based CAR show potent MMP-2–dependent cytotoxicity in glioblastoma models. We hypothesized that affinity maturation of CTX via phage display could enhance MMP-2 binding and improve CAR T-cell performance.

**Methods:**

A CTX-based phage display library (∼1.3 million variants) was screened against immobilized MMP-2. A lead variant, CTXA8, was recombinantly expressed and tested for binding specificity against a panel of off-target proteins. Its cellular uptake and localization were evaluated, followed by incorporation into CAR constructs in both single-unit and tandem formats (eCTXA8-CAR). The cytotoxicity of CTX-, CTXA8-, and eCTXA8-CAR T cells was assessed against glioblastoma cell lines and primary patient-derived tumor cells.

**Results:**

Screening identified CTXA8, which demonstrated a 4.4-fold increase in MMP-2 affinity and reduced off-target binding relative to CTX. Fluorescent CTXA8 showed 2.4–3.5-fold greater uptake in glioblastoma cells than CTX. Among the CAR constructs tested, eCTXA8 induced the highest IFN-γ release. eCTXA8-CAR T-cells consistently outperformed CTX-CAR and non-transduced T cells in cytotoxicity assays, especially at low effector-to-target ratios.

**Conclusion:**

This study demonstrates that directed evolution of CTX can produce high-affinity, selective MMP-2 ligands suitable for next-generation CAR T-cell therapies. CTXA8-based CARs offer enhanced anti-tumor efficacy, supporting their potential in overcoming challenges in solid tumor immunotherapy.

## Introduction

Miniproteins, also known as cystine-dense peptides, are compact biomolecules (typically <10 kDa or <40 amino acids) that have garnered significant interest for drug development. These peptides lack a hydrophobic core and instead they stabilize their tertiary structure through extensive disulfide bonding. Over 100,000 naturally occurring variants have been identified across diverse taxa (Baker et al., 2017), forming scaffold families with shared structural motifs. Miniproteins combine the benefits of small molecules and large biologics due to their distinctive stability, binding specificity, and low immunogenicity (Crook et al., 2020; Dardevet et al., 2015). The versatility of miniproteins extends beyond their native form. Advances in protein engineering and directed evolution systems—such as phage and yeast display—enable the generation of miniprotein analogues with enhanced binding affinity and specificity towards a wide variety of targets. These technologies facilitate the construction of megalibraries via random mutagenesis or employ more sophisticated approaches such as recombination of homologous segments of miniproteins from the same scaffold family (Takacs et al., 2009). This approach can maximize the ratio of correctly folded variants in the library. Through iterative rounds of selection, high-performance binders against specific molecular targets can be isolated.

Chlorotoxin (CTX), a 36-amino-acid miniprotein originally isolated from the venom of the *Leiurus quinquestriatus* scorpion, illustrates the potential of naturally derived peptides as precision-targeting agents in cancer therapy. CTX exhibits selective binding to glioma and other tumor cells, with minimal affinity for normal brain tissue, making it a promising candidate for glioblastoma (GB) targeting (Dastpeyman et al., 2019; Shrestha et al., 2022). More broadly, scorpion venoms contain diverse bioactive peptides with emerging applications in oncology, including tumor targeting and anticancer therapy, further supporting the therapeutic relevance of venom-derived miniproteins (El-Qassas et al., 2024; El-Qassas et al., 2025). While early studies linked its tumor specificity to interactions with the chloride channel CLC3 (DeBin et al., 1993), more recent research has identified additional binding partners, including matrix metalloproteinase-2 (MMP-2), neuropilin-1 (NRP1), and annexin A2 (ANX2) (Deshane et al., 2003; Kesavan et al., 2010; McGonigle et al., 2019). Notably, MMP-2 has emerged as the most functionally relevant target due to its overexpression in up to 80% of GB tumors and its essential role in extracellular matrix degradation and tumor invasion (Kolli-Bouhafs et al., 2012; Lu et al., 2004; Nakada et al., 1999). Despite previous unsuccessful attempts to selectively inhibit MMP-2 pharmacologically due to its homology with other MMPs (Farkas et al., 2023; Laronha and Caldeira, 2020; Proenca et al., 2019; Wang D. et al., 2020; Yano et al., 2020), its surface accessibility and differential expression continue to make it a compelling therapeutic target. Indeed, CTX binds to MMP-2 with submicromolar affinity (Farkas et al., 2023), providing a foundation for engineering improved variants.

Engineered miniproteins like CTX and its analogues have been applied across a range of biomedical platforms (Dardevet et al., 2015) including tumor imaging (Yamada et al., 2021), drug delivery, and immunotherapy. A major application of miniproteins is their integration into chimeric antigen receptor (CAR) constructs as tumor-targeting domains. CARs are synthetic receptors engineered into immune cells, most commonly T-cells, to recognize tumor antigens through an extracellular binding domain linked to intracellular signaling domains such as CD3ζ, CD28, or 4-1BB that activate T-cells upon antigen binding (Prommersberger et al., 2020; Uscanga-Palomeque et al., 2023). This modular architecture enables CAR T-cells to recognize and kill target cells independently of MHC presentation, offering a powerful approach for treating cancers, particularly hematological malignancies such as B-cell leukemias and lymphomas (Shrestha et al., 2022; Awasthi et al., 2023; Lu et al., 2022). However, their application to solid tumors like glioblastomas has been limited by tumor antigen heterogeneity, immunosuppressive microenvironments, and off-target toxicities.

Unlike traditional scFvs, which can suffer from folding instability or immunogenicity, engineered miniproteins such as CTX offer robust expression, compact size, and favorable biophysical properties. CTX-based CAR T-cells have demonstrated potent and selective cytotoxicity against GB models in preclinical studies, with efficacy dependent on MMP-2 expression on tumor cells (Wang D. et al., 2020). These findings underscore the potential of directed evolution to transform native peptides into next-generation immunotherapeutic agents.

In our study, we hypothesized that optimizing CTX via phage display would yield an analogue with enhanced affinity and selectivity for MMP-2. This led to the development of CTXA8, a novel recombinant miniprotein confirmed through fluorescent labeling and cellular assays to maintain specific MMP-2 binding (Farkas, 2024). Capitalizing on this affinity, we incorporated CTXA8 into a CAR framework for T-cell redirection. CAR T-cell therapy, although transformative for hematological malignancies such as acute lymphoblastic leukemia, diffuse large-B cell lymphoma, and chronic lymphocytic leukemia (Park et al., 2018; Schuster et al., 2019; Wang M. et al., 2020), has struggled to demonstrate the same efficacy in solid tumors like GB. Limitations stem from antigen heterogeneity, immunosuppressive microenvironments, and lack of tumor-specific targets (Prommersberger et al., 2020; Uscanga-Palomeque et al., 2023).

Beyond designing single CTX and CTXA8 epitope presenting CAR constructs, we also created a dual-epitope construct containing tandem CTXA8 units (‘enhanced-’ eCTXA8-CAR) to increase avidity. The tandem design was motivated by previous findings suggesting that multivalent ligand configurations in the extracellular domain can augment T-cell activation through cooperative binding effects (Chaudhary et al., 2018). When tested *in vitro* against glioblastoma cell lines and patient-derived primary GBM cells, these CAR T-cells demonstrated enhanced cytolytic activity, underscoring the potential of rationally evolved miniproteins for precision cell therapy applications.

In summary, our work illustrates how *de novo* miniprotein engineering can yield high-affinity ligands for integration into next-generation CAR constructs, potentially overcoming key limitations of solid tumor immunotherapy.

## Materials and methods

### A combinatorial miniprotein library of CTX

For megalibrary design, 28 unique homologous miniproteins (including CTX) were identified through an extensive BLAST search of the UniProt database (UniProt, 2023). These molecules belong to the knottin scaffold family, characterized by a cystine-knot topology with disulfide connectivity 1–4, 2–6, 3–7, 5–8 (Correnti et al., 2018; Supplementary Table S1). Based on homology and feasibility considerations, the amino acid sequences of the chosen miniproteins were divided into three segments (denoted A, B, and C). The segments were linked by sharing nucleotide codes for the third cysteine (segment A and B) and for the sixth cysteine (segment B and C). Segments were designed around conserved cysteine positions to preserve the cystine-knot topology while maximizing sequence diversity within structurally permissive regions. To increase library size, all segments were further divided into two subsegments. The resulting subsegments were combined to create 121, 94, and 121 unique A, B and C segments, yielding a phage library with a diversity of 1,376,254 variants. The library creation was as follows: first, complementary nucleotide pairs for each unique segment were phosphorylated by T4 polynucleotide kinase (EK0031, Thermo Fisher), following the manufacturer’s protocol. The phosphorylated oligos were annealed by heating the samples to 95 °C, then they were tapered slowly to 24 °C. Oligo duplexes coding for segments A and C were mixed in equimolar amounts, and this “supermix” was added to separate ligation reaction tubes, each containing a unique segment B coding oligo duplex to improve successful library assembly. The segments were cloned into a linearized pAS62 phagemid vector (Farkas, 2024) using T4 DNA ligase (EL0013, Thermo Fisher), following standard protocol. The final phagemid construct harbored a signal sequence followed by a member of the miniprotein library, a linker sequence (GSASSATR), and the C-terminal part of the P3 coat protein (amino acids: 216–424 of NP_510891.1). The next day, prior to electroporation, to increase transformation efficiency, ligation mixtures were pooled and purified using the NucleoSpin Gel and PCR Cleanup Kit (Cat. No. 740609, Macherey Nagel) according to the manufacturer’s instructions. Next, purified ligation products were transformed into SS320 electrocompetent cells (60514-1-LU, BioCat), then the cell culture was infected with M13KO7 helper phage (N0315S, New England Biolabs). Phage propagation and preparation followed a standard protocol (Tonikian et al., 2007). The particle concentrations of phage solutions were determined using a NanoDrop One C (Thermo Scientific) spectrophotometer. The phage particle concentration was assessed according to the formula (OD_268_-OD_320_) × 5 × 10^12^ particles/mL (Tonikian et al., 2007). Phage stock solution was stored at 4 °C, in phage resuspension buffer (TBS, 0.05% w/v Tween-20, 5 mg/mL BSA). The quality of the phagemid library was assessed by next-generation sequencing (NGS), which confirmed the presence of more than 1 million miniprotein variants of the theoretical 1.3 million members of the library.

### Library screening by phage display

Eight wells in a NUNC-Immuno MaxiSorp 96-well plate (430,341, Thermo Fisher) were coated overnight at 4 °C with 0.5 µg (100 µL in ultrapure water) of recombinant human MMP2 (SRP3118, Sigma Aldrich) per well. The same number of wells were blocked with 200 μL blocking buffer (TBS, 5 mg/mL BSA), serving as negative control. Next day, the wells were emptied, then blocked for 1 h at room temperature (RT) with 200 μL blocking buffer, then washed 4 times with washing buffer (TBS, 0.05% w/v Tween-20). 100 μL CTX phage library solution (phage titer: 10^12^ cfu/mL, in phage resuspension buffer) was added to the wells and was incubated for 3 h at RT, after which the wells were washed 12 times with 300 μL washing buffer, then 100 μL 100 mM HCL was added and incubated for 5 min to elute the bound phages. After adjusting the pH to 7.0–8.5 by neutralization solution (1 M Tris), the phage supernatants from the MMP2 wells were used to infect XL1-Blue *E. coli* cells (200,236, Agilent), to re-amplify the library. After three rounds of panning cycle, the DNA sequence of the individual peptide clones displayed by phages was identified by Sanger sequencing.

### Recombinant expression of CTX and CTXA8

For the recombinant production of CTX and CTXA8 miniproteins, the corresponding DNA sequences were cloned into a pET-based expression vector. The expression construct contained an N-terminal 6×His-tag followed by the prokaryotic disulfide bond isomerase DsbC to facilitate proper folding, a WELQ recognition site for the staphylococcal serine protease SplB, and the C-terminally cloned miniprotein coding sequence. After expression and purification, the DsbC fusion partner and the 6×His-tag were removed by site-specific proteolytic cleavage and subsequent chromatographic purification steps. Consequently, the final CTX and CTXA8 preparations used in binding and functional assays did not contain residual His-tags. Sequence-verified plasmids were transformed into SHuffle T7 Express cells (C3029, New England Biolabs) and streaked onto LB agar plates supplemented with 100 μg/mL carbenicillin (APOSBIC0109, VWR Chemicals). The following day, a single colony was inoculated into 50 mL of LB medium containing 100 μg/mL carbenicillin and cultured at 30 °C with shaking at 200 rpm for 16 h. Subsequently, 5 mL of the overnight starter culture was transferred into each of two flasks containing 1 L of LB medium supplemented with 100 μg/mL carbenicillin and incubated at 37 °C with shaking at 200 rpm. When the optical density at 600 nm (OD600) reached 0.6–0.8, the cultures were cooled to 28 °C and protein expression was induced with 0.5 mM IPTG (4,371,45X, VWR Chemicals) for 16 h. Cells were harvested by centrifugation at 4,000 *g* at 4 °C for 15 min, and the resulting cell pellets were resuspended in 2 × 30 mL of lysis buffer (PBS, pH 7.4, supplemented with Triton X-100% and 10% Sigma Fast protease inhibitor cocktail solution (S8830, Sigma)) and stored at −20 °C.

### Purification of CTX and CTXA8

Frozen cells were thawed at RT, then disrupted by ultrasound, and the cell debris was pelleted by centrifugation at 20.000 g for 10 min at 4 °C. The supernatant (S/N) was transferred into a clean centrifuge tube and centrifuged again, then filtered through a 0.22 μm PS filter (GPWP04700, Millipore express). 2 × 5 mL HisTrap™ High Performance Ni columns (GE17-5248-01, Sigma) connected in tandem were pre-equilibrated with five column volume (CV) (50 mL) PBS, then the filtered S/N was loaded using a peristaltic pump at 2 mL/min flow rate. Loaded columns were connected to an Äkta Pure (77120-62, Cytiva) chromatography system. After washing with PBS, bound material was eluted using a linear gradient (0%–100%, in 10 CV) with IMAC Buffer A (PBS, pH 7.6) and IMAC Buffer B (PBS, pH 7.4, 500 mM imidazole) at a flow rate: 2 mL/min. Eluted fractions containing the fusion protein were pooled and diluted with HEPES dilution buffer to 3 times volume. The pooled material was concentrated back to the initial volume using an AMICON Stirred Cell equipped with 10 kDa MWCO membrane (UFSC05001, Merck). This step was repeated 2 times. The dialyzed sample was digested by SplB protease at RT for 16 h to remove the DsbC fusion partner.

The digested product was centrifuged for 10 min at 3000g, then filtered with a 0.22 µm syringe filter, then loaded onto an IEX HiScreen™ SP High Performance column (GE28-9,505-15, Merck) pre-equilibrated with IEX Buffer A (PBS, pH 7.4), using a peristaltic pump at 0.5 mL/min flowrate. The loaded column was connected to an Äkta pure chromatographic system. Following 5 CV washing with PBS, the material was eluted with a linear gradient (0%–50%) with IEX Buffer A and IEX Buffer B (PBS, 1M NaCl) at 0.6 mL/min flow rate, and 5 mL fractions were collected.

Fractions containing the product were pooled and supplemented with ¼ volume of HPLC Eluent A (Acetonitrile). The solution (maximum 5 mL/HPLC run) was injected into a Jasco HPLC system with a Teknokroma Protein C4 column (TR-010118, ABL&E-JASCO) connected. The protein was purified using the following gradient: 0 min: 0% HPLC Eluent B (Acetonitrile, formic acid), 2 min: 0% B; 15 min: 20% B; 25 min: 25% B with 1.2 mL/min flow rate at 40 °C. Fractions were analyzed by analytical HPLC, and protein containing fractions were pooled. Protein concentration was determined by analytical HPLC, and the material was then lyophilized and stored at −80 °C.

### Cy5 labeling of CTX and CTXA8

CTX-Cy5 and CTXA8-Cy5 were synthesized at Vichem Chemie Research Ltd. By conjugation reaction of CTX and CTXA8 with cyanine 5 N-hydroxysuccinimide ester (Lumiprobe) (Farkas, 2024). The reacted samples were purified to homogeneity using RP chromatography (RP-HPLC–UV/MS) by collecting the fractions of a peak containing monoconjugated CTX-Cy5 and CTXA8-Cy5. According to structure elucidation studies by analytical RP-HPLC followed by tandem mass spectrometry, the CTX-Cy5 product used in the present studies proved to be a mixture of monoconjugates labeling the three lysine residues at positions 15, 23, and 27 in approximate amounts of 20%, 38%, and 42%, respectively. As CTXA8 harbors only one lysine at position 26, the labeled compound is a single product. Mass spectrometric analysis confirmed exclusive monoconjugation of both CTX-Cy5 and CTXA8-Cy5, and no multi-labeled species were detected in the purified preparations used for functional assays (Farkas, 2024).

### Ig-coated bead test

Ig-coated bead tests were performed as described previously (Farkas et al., 2023). Briefly IgG coated magnetic beads (Magnabind™ goat, anti-rabbit, 21,356, Thermo Fisher Scientific) were incubated with anti-MMP-2 antibody in 1:100 dilution (rabbit IgG, HPA001939) for 1 h at 4 °C. Subsequently, 10 μg/mL MMP-2 (SRP3118, Sigma) protein was added to the beads and incubated overnight at 4 °C. For the fluorescent labeling 1 µM Cy5-conjugated CTX and CTXA8 were applied.

For competitive displacement experiments, the target protein–coated beads were resuspended in 50 μL test solution containing the specified concentration of the unlabeled test substance (CTX or CTXA8) dissolved in DPBS and incubated for 1 h at RT. Subsequently, 50 μL DPBS solution containing 2 μM Cy5 labeled CTX, and the specified concentration of the unlabeled test substance were added to incubate the beads in 1 μM labeled CTX (displaced indicator ligand) and the specified concentration of the unlabeled test substance (displacing ligand). The beads were incubated for an additional 1 h at RT, then washed and magnetically separated three times, resuspended in 2 mL DPBS, and were ready for flow cytometry measurement.

Fluorescent signals were analyzed by flow cytometry with a MACSQuant 10 flow cytometer (Miltenyi Biotec). Binding of labeled ligand was determined by median fluorescent intensity of gated events compared to negative control beads (without target proteins) out of 10.000 recorded events for each test.

### Assessment of binding of labeled CTX and CTXA8 to various target proteins by the cobalt-coated bead test

CTX-Cy5 and CTXA8-Cy5 binding intensity to target proteins was analyzed by the Cobalt-coated bead test using Dynabeads™ His-Tag Isolation and Pulldown (ThermoFisher Scientific) as described previously (Farkas et al., 2023). The test is based on the principle of anchoring various 6xHis-tagged target proteins (receptors) to immobilized cobalt-coated His-Tag Isolation and Pulldown Dynabeads (ThermoFisher Scientific), then binding a fluorophore labeled ligand–here CTX-Cy5 or CTXA8-Cy5 – to the target protein and measuring the bead-bound fluorescence intensity by flow cytometry. Briefly beads were washed three times with Tris Buffered Saline solution (TBS, Thermo Fisher Scientific) containing 0.05% (v/v) Tween-20 and incubated overnight at 4 °C with the selected target proteins in 0.16 µM concentration, which is equivalent to 10 μg/mL active MMP-2 (MW 62 kDa). The used target proteins were the following (with NCBI accession number [segment amino acid sequence]; His-tag position: C- or N-terminal; supplier; and catalog number# listed in parentheses): MMP2 (NP_004521.1 [110–660]; N-; ProSpec, #ENZ 769), NRP1 (NP_001019799.2 [22–644]; C-; Sino Biological; #10011-H08H); MMP-9 (NP_004985.2 [20–701]; C-; ProSpec; #ENZ 1091), TIMP-2 (NP_003246.1 [27–220]; N-; ProSpec; #ENZ-646), MMP 14 (NP_004986 [24–524]; C-; ThermoFisher; #RP77533), ANX2 (NP_001002857.1 [1–339]; N-; ProSpec; #PRO-777), αvβ3 integrin (AAA52589.1 and NP_002196.4 heterodimer; both with C-terminal His-tag; Native Antigen Company; #REC31719-100), CLC-3 (NP_001820.2 [1–818]; C-; Creative Biomart; custom made by recombinant expression in *E. coli*), human serum albumin (NP_000468.1 [25–609]; C-; Abcam; #ab217817). Following target protein incubation, the beads were washed three times and then blocked by incubation with 100 µL Casein Blocking Solution (B6429, Sigma) for 1 h at RT, followed by three rounds of washing. Then the beads were incubated with 1 µM Cy5 conjugated CTX or CTXA8 for 1 h at RT.

Fluorescent signal was analyzed by flow cytometry with MACSQuant Analyzer 10. Binding was determined by median fluorescent intensity compared to negative control beads (without target proteins) out of 10.000 recorded events.

### Confocal imaging

U251-MG cells were seeded into eight well Ibidi® μ-Slide microscopic slides (Ibidi GmbH, Gräfelfing, Germany) and let adhere for 48 h. Cell culture medium was replaced by treating medium. Treating medium contained 0.5 µM CTXA8-Cy5, LysoView 488 fluorescent probe (70067, Biotium) in 1,000x dilution and CellBrite Steady 550 fluorescent probe (30107, Biotium) in 500x dilution. Cells were treated for 90 min at 37 °C in a CO_2_ incubator after that treating medium was removed and replaced with ice-cold 5% FBS-containing PBS. Images were acquired with a Zeiss Confocal LSM 710 microscope (Carl Zeiss AG, Oberkochen, Germany). Objective: Plan-Apochromat ×63/1.40 Oil DIC M27. Pinhole: 1.0 AU. Laser wavelengths were 488 nm, 543 nm and 633 nm. Detector wavelengths were 490–533 nm, 593–622 nm and 661–730 nm. Images were processed by the software Zeiss ZEN Lite (version 2.3).

### Flow cytometric analysis of Cy5-labeled CTX and CTXA8 cellular uptake

Tested cell lines were incubated (double stained) with 2.5 µM viability marker (Po-Pro-1 dye) and CTX-Cy5 or CTXA8-Cy5 in 300 nM concentration. The Cy5-conjugated protein uptake was measured as the increase in median fluorescence intensity (ΔMFI) relative to Cy5-protein unlabeled cells, after gating out non-viable cells based on the Po-Pro-1 staining. A set of representative tumor cell lines were included in this study, comprising human glioblastoma (U251-MG) pancreatic ductal carcinoma (Panc-1), melanoma (A375) and breast cancer (SK-Br) cells, which have been classified as ‘CTX positive’ in previous studies (Lyons et al., 2002). Additionally, normal non-tumor cells, i.e., human dermal fibroblasts (HDFa) were also investigated to reveal alterations in tumor selectivity.

### Design and creation of CAR constructs

The CTX, CTXA8 and the duplicated CTXA8 (eCTXA8) chimera antigen receptor (CAR) constructs were designed using the SnapGene® software (from Dotmatics). All constructs consisted of the IgG heavy chain signal peptide, the miniproteins CTX, CTXA8, or the double CTXA8 connected by a short linker sequence, the CD8 hinge, the transmembrane region of human CD28, and the cytoplasmic region of human CD3 zeta, with CD28 intracellular costimulatory endodomains (see Supplementary Figure S1). A truncated CD19 was incorporated to allow tracking of transduced cells. The coding sequences of CTX and CTXA8 were purchased from Eurofins Genomics and were inserted into pSFG retroviral vectors at NcoI and KasI restriction sites. The CAR plasmids were analyzed by Sanger sequencing with primers covering the CD8h hinge region (data not shown).

### Retrovirus production and transduction of T-cells

RD114 pseudotyped retroviral particles were generated by transient transfection of HEK 293T-cells with the CTX/eCTXA8 CAR-encoding pSFG retroviral vectors, the Peg-Pam-e plasmid coding MoMLV gag-pol, and the pMax. RD114 plasmid using jetPrime transfection reagent (Polyplus, Illkirch, France). Supernatants containing the retrovirus were collected after 48 h.

To generate CTX and eCTXA8-redirected CAR T-cells, human peripheral blood mononuclear cells were isolated by Ficoll gradient centrifugation and stimulated in non-tissue culture 24-well plates precoated with 1 μg/mL anti–CD3 OKT3 (ThermoFischer, Waltham, MA, USA) and anti-CD28 (R&D Systems, Minneapolis, MN, USA) antibodies. On day 2, human interleukin-2 (IL2; 50 IU/mL) (Proleukin, Clinigen, Yardley, PA, USA) were added to cultures. T-cells were transduced with retroviral particles on RetroNectin-coated (Takara, Kusatsu, Japan) plates on Day 3 in the presence of IL2 (50 IU/mL). The expansion of T-cells was subsequently supported with cytokines. OKT3/CD28 activated non-transduced (NT) T-cells were expanded with the above-described cytokines using the same protocols. After 48 h of incubation, the cells were used for further experiments. Experiments on human samples were carried out in accordance with the Declaration of Helsinki and approved by the Regional and Institutional Committee for Research Ethics (RKEB.5378/2019).

### Flow cytometric confirmation of CAR expression

CTX-, CTXA8-and eCTXA8-directed CAR expression was confirmed by flow cytometric analysis using an APC-conjugated anti-human CD19 antibody (BD Biosciences, San Jose, CA, USA). Briefly, transduced T cells were harvested 72 h post-infection, washed twice with ice-cold PBS, and incubated for 10 min at 4 °C with the APC-conjugated anti-human CD19 antibody, which binds to the truncated CD19 tag encoded in the CAR construct as a surface expression marker. Following staining, cells were washed and resuspended in PBS for acquisition on a NovoCyte (ACEA Biosciences, San Diego, CA, USA) flow cytometer. Data were analyzed using NovoExpress software (ACEA Biosciences, San Diego, CA, USA). Expression of the CAR was quantified as the percentage of CD19-positive cells within the CD3^+^ lymphocyte gate, and transduction efficiency was consistently observed to exceed 75% across independent experiments.

### Western blot analysis of CAR expression

T cells transduced with CTX-CAR, CTXA8-CAR, eCTX-CAR, or eCTXA8-CAR constructs, as well as non-transduced (NT) control T cells, were harvested and lysed in RIPA buffer supplemented with protease inhibitors. Equal amounts of total protein were separated by SDS-PAGE under non-reducing conditions and transferred to PVDF membranes. Membranes were blocked with 5% non-fat milk in TBS-T and incubated overnight at 4 °C with an anti-CD3ζ primary antibody. After washing, membranes were incubated with HRP-conjugated secondary antibodies and developed using enhanced chemiluminescence. Signals corresponding to endogenous CD3ζ and CAR-associated CD3ζ fusion proteins were visualized and documented using a chemiluminescence imaging system.

### Cells and culture conditions

#### Cell lines

HEK 293 T packaging cells and U251 human glioblastoma cell lines were purchased from the American Type Culture Collection (ATCC, Manassas, VA, USA). Cells were cultured in Dulbecco’s Modified Eagle Medium (DMEM) supplemented with 2 mM/L GlutaMAX and 10% Fetal Bovine Serum (FBS) and antibiotics. Primary human T-cells and CAR T-cells were cultured in RPMI (Roswell Park Memorial Institute) medium supplemented with 2 mM/L GlutaMAX, 10% FBS and antibiotics.

#### Primary GBM cells

Primary GBM cells were isolated from samples kindly provided by László Sipos and Imre Fedorcsák originated from both male and female patients with the median age of 61 years old. Excised tumors were processed within 2 hours after surgery. Briefly, tumor mass was washed twice with 1x PBS and minced to approximately 1 mm^3^ parts. For the elimination of excess red blood cells ACK Lysis Buffer (A1049201, Thermo Fisher Scientific, Waltham MA, USA) was applied for 2 min and washed again with 1x PBS followed by centrifugation. The tissue pellet was resuspended in Tryple E (12605010, Thermo Fisher Scientific, Waltham MA, USA) and cells were enzymatically digested for 30 min at 37 °C, followed by inactivation with 20% FBS containing Minimal Essential Media (M2279, Sigma-Aldrich). Cells then were transferred to Petri dishes and cultured. For the functional assays with CAR T-cells and gene expression analysis, cell cultures below passage four (P4) were used.

### Characterization of primary GBM cells

Gene expression of selected targets, including cellular tumor antigen p53 (*TP*53), cyclin-dependent kinase inhibitor 2A (*CDKN2A*) and phosphatidylinositol-4,5-bisphosphate 3-kinase catalytic subunit α (*PIK3CA*), epidermal growth factor receptor (*EGFR*), neurofibromin-1 (*NF1*), *MMP-2* and beta actin (*ACTB*) was analyzed by quantitative PCR (Ah-Pine et al., 2023; Heimberger et al., 2005; Janik et al., 2019; Lee et al., 2020; Zhao et al., 2017). Primer sequences are provided in Supplementary Table S2. Total RNA was isolated from primary GBM cells (S7, S8, S10, S12), U251-MG and T98G cell lines, and HEK293T control cells. From each cell type 500 ng RNA was transcribed to cDNA using High-Capacity cDNA Reverse Transcription Kit (Applied Biosystems™, Thermo Fisher Scientific, Waltham MA, USA). Gene expression was analyzed by QuantStudio™ 5 system (Thermo Fisher Scientific Waltham MA, USA) and comparative ΔΔCt analysis performed with ACTB as a reference gene and HEK293T-cells as a reference sample.

### Cytokine secretion assay

CTX, CTXA8 or eCTXA8 CAR T-cells were plated onto 1 μg/mL MMP-2 pre-coated plates in 1 × 10^5^ cell number or cocultured with glioblastoma cell lines at a 1:1 effector to target ratio. Following 24 h of culturing, the supernatant was harvested and analyzed for the presence of interferon-gamma (IFN-γ) by ELISA (R&D systems, Minneapolis, MN, USA) according to the manufacturer’s instruction using a Spark® multimode microplate reader (Tecan Group Ltd., Männedorf, Switzerland). Medium and NT T-cells served as controls.

### Potency assays with U251, T98G and primary GBM target cells

Target cells (U251, T98G, and primary glioblastoma cells) were seeded in 96-well plates at a density of 3 × 10^4^ cells per well and allowed to adhere overnight. The following day, target cells were co-incubated with varying numbers of CTX- or eCTXA8-CAR T cells, as well as non-transduced control T cells, to achieve effector-to-target (E:T) ratios ranging from 0.003:1 to 10:1. Co-cultures were maintained in the presence of recombinant human IL-2 (50 U/mL) to support T-cell viability.

After 24 h of co-culture, both suspension cells and adherent target cells (detached using trypsin) were collected, combined, and washed with PBS. Cells were then stained with CD45-APC (final concentration 5 μg/mL, 10 min on ice) to distinguish effector T cells from target tumor cells. Target cell viability was assessed by flow cytometry using a NovoCyte instrument (ACEA Biosciences, San Diego, CA, USA), and data were analyzed with NovoExpress software.

At least 10,000 events were acquired per sample. The gating strategy first identified the main cell population based on forward scatter (FSC) and side scatter (SSC) characteristics to exclude debris and aggregates. Within this morphological gate, CD45-negative cells were defined as target tumor cells, and viability was quantified accordingly. Target cell viability was expressed as a percentage relative to non-treated target cells cultured under identical conditions without effector T cells, which served as the reference control.

### Institutional review board statement

All procedures were performed after obtaining informed consent and following IRB (Institutional Review Board)-approved protocols at the Department of Neurosurgery and Neurointervention, Semmelweis University, Budapest, Hungary. Our research was conducted according to the principles of the Declaration of Helsinki and approved by the Hungarian Scientific and Research Ethics Committee of the Medical Research Council (ETT TUKEB; Decree No. BM/9,204- 1/2023).

### Statistical analysis

GraphPad Prism 8 software (GraphPad software, Inc., La Jolla, CA) was used for statistical analysis. Data are presented as mean ± SD or ±SEM for the *in vivo* studies. For comparison between two groups, a two-tailed t-test was used. For comparisons of three or more groups, one-way ANOVA with Bonferroni’s *post hoc* test was used.

## Results

### Selection of improved CTX analogues by biopanning of a phage display miniprotein library

**FIGURE 1:**
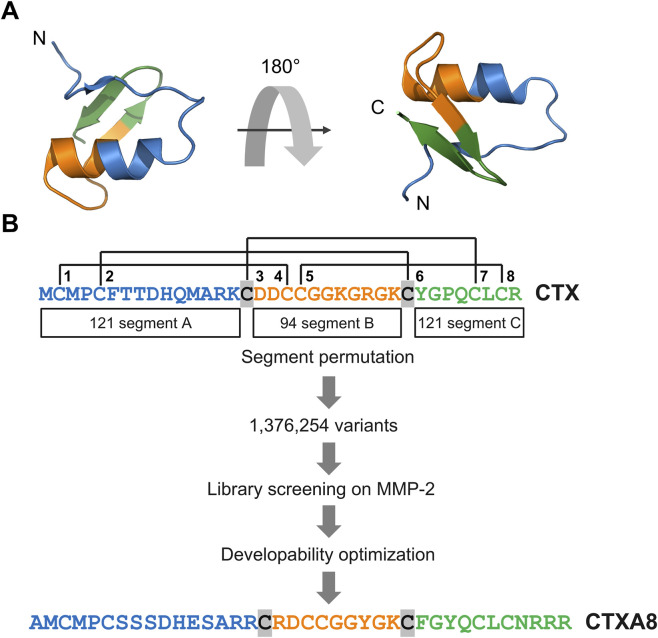
Schematic overview of chlorotoxin based megalibrary design. **(A)** Structure of chlorotoxin (CTX), shown in backbone representation (PDB accession number: 6ATW). Blue, orange and green coloring mark the artificial segment design for miniprotein library assembly. **(B)** Combinatorial library design of CTX homologues. 28 unique native toxin peptide sequences belonging to the same scaffold family were chosen for library design (for additional information, see Supplementary Table 1). Based on homology considerations, the amino acid (aa) sequence of CTX and its homologues were divided into three segments (marked with blue, orange and green for segment A, B and C, respectively). Segments were linked by sharing nucleotide codes for the third and sixth cysteine, highlighted with grey in the CTX aa sequence. Each segment was further divided into two subsegments, which were combined to create 121, 94, and 121 unique segments for A, B and C, respectively, yielding a phage library with a diversity of 1,376,254. Disulfide pattern is indicated, and the numbering of the cysteine residues is also highlighted in the aa sequence of CTX.

Our initial goal was to develop a CTX analogue with enhanced affinity and selectivity for MMP-2 using phage display. The library construction and screening process are summarized in Figure 1. The phage library was screened on immobilized recombinant human MMP-2 protein in a multiwell plate format. The enrichment of promising mutants was monitored by Sanger sequencing, and binders were confirmed by bead binding assays (see Materials and Methods, and (Farkas et al., 2023)). Phage-displayed CTX analogues were ranked based on their MMP-2 binding affinity, and an analogue with the highest affinity was selected as the lead candidate. Its sequence was optimized to enhance developability by mutating specific amino acids to improve solubility and creating a monolysine variant to facilitate labeling via the ζ-amino group of lysine at position 26. This improved analogue was designated as CTXA8. The details of this lead selection process were described previously in a patent application (Farkas, 2024).

### CTXA8 demonstrated improved affinity and selectivity towards MMP-2

**FIGURE 2:**
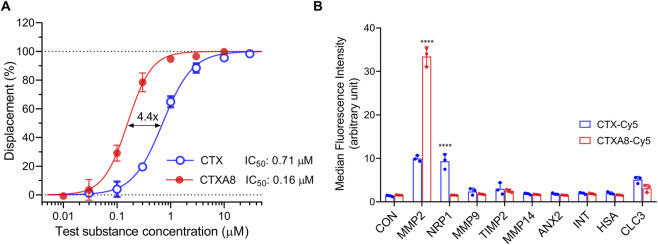
CTXA8 binds to MMP-2 with higher affinity and selectivity than CTX. **(A)** Concentration-displacement relationships for CTX and CTXA8 against 1 µM CTX-Cy5 as displaced ligand in the Ig-coated bead test (Farkas et al., 2023) indicating the difference between their affinities to MMP-2 protein. The results are presented as mean ± SD of two independent experiments comparing the two compounds, and sigmoidal curve fitting on the mean values. **(B)** Binding profile of CTXA8-Cy5 and CTX-Cy5 to a panel of potential target proteins. Abbreviations: control (CON), matrix metalloproteinase-2 (MMP-2), neuropilin-1 (NRP1), matrix metalloproteinase-9 (MMP-9), tissue inhibitor of metalloproteinase-2 (TIMP-2), matrix metalloproteinase-14 (MMP-14), annexin 2 (Anx2), αVβ3 integrin (INT), CLC3 (chloride ion channel) and human serum albumin (HSA). The bar chart represents mean ± SD of 3 separate experiments with superimposed scatter plot. *** indicate a statistically significant difference (p < 0.001) from control (ANOVA followed by Dunnett’s test).

To characterize CTXA8 in comparison with CTX, both miniproteins were produced recombinantly. The MMP-2 binding affinities of CTXA8 and CTX were evaluated by measuring the concentration dependent displacement of CTX-Cy5 (1 µM) by the unlabeled miniproteins. In the Ig-coated bead test. CTXA8 demonstrated a 4.4-fold higher affinity for MMP-2 compared to the original CTX (Figure 2A). The binding profiles of CTX-Cy5 and CTXA8-Cy5 were compared across a panel of proteins identified in the literature as potential binding partners of CTX (Cohen et al., 2018; Fang et al., 2010; Qin et al., 2014; Wang et al., 2013). Control beads (CON) were only coated with the blocking agent casein. The tested proteins included matrix metalloproteinase-2 (MMP-2), neuropilin-1 (NRP1), matrix metalloproteinase-9 (MMP-9), tissue inhibitor of metalloproteinase-2 (TIMP-2), matrix metalloproteinase-14 (MMP-14), annexin 2 (Anx2), α_V_β_3_ integrin (INT), CLC3 (chloride ion channel 3), and human serum albumin (HSA) as a non-target protein control (Figure 2B). The results showed varying binding intensities of CTX-Cy5 and CTXA8-Cy5 (both applied at 1 µM concentration) to the different postulated target proteins, as measured by bead-bound fluorescence intensities in bead binding tests. At the tested concentration, CTX-Cy5 showed similar binding intensity to MMP-2 and NRP1, weaker but detectable binding to MMP-9, TIMP-2, and CLC3, and negligible binding to MMP-14, α_V_β_3_ integrin, Anx2, and HSA. In contrast, CTXA8-Cy5 bound to MMP-2 with at least threefold higher signal intensity compared to CTX-Cy5, while its binding to all other proteins in the panel, except CLC3, was negligible. These results indicate that CTXA8 not only adopts the anticipated cystine-stabilized tertiary structure, but also exhibits improved binding intensity and selectivity for MMP-2.

### Cancer cell lines exhibited higher uptake of CTXA8-Cy5 compared to CTX-Cy5

Comparative uptake studies of Cy5-labeled CTX and CTXA8 were conducted on various cancer cell lines to assess enhanced cancer cell targeting. Glioblastoma (U251-MG), melanoma (A375), pancreatic carcinoma (Panc-1), and breast carcinoma (SK-Br) cell lines, as well as non-cancerous fibroblasts (HDFa), were incubated with 300 nM Cy5-labeled CTXA8 or CTX for 45 min at 37 °C. Fluorescence intensity was subsequently measured by flow cytometry.

**FIGURE 3:**
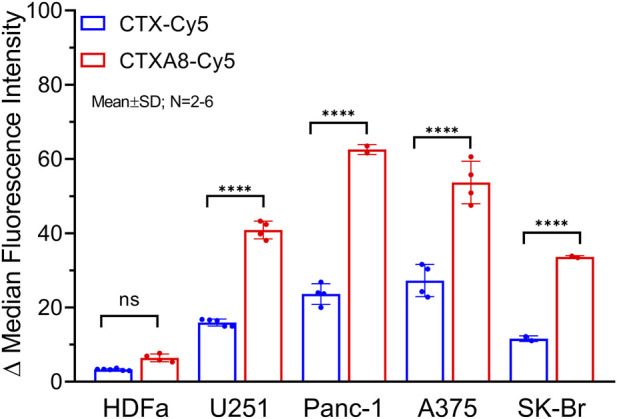
Cellular uptake of Cy5 conjugated CTX and CTXA8. Fluorophore labeled ligands in the cell staining intensity test as measured by flow cytometry. 300–300 nM Cy5 conjugated CTX and CTXA8 miniproteins were incubated with selected cell lines: the non-cancerous HDFa, and the cancerous U251-MG, Panc-1, A375 and SK-Br cell lines. The data are presented as mean ± SD of N = 2 (CTX-Cy5 samples) or six experiments (CTXA8-Cy5 samples) comparing the staining intensity of the two compounds in 5 cell lines. ****p < 0.0001.

The results showed that relatively large quantities of labeled CTX and CTXA8 were taken up by the ‘CTX-positive’ tumor cells, while uptake was much lower in the ‘normal’ fibroblast cells. The uptake of CTXA8-Cy5 was higher across all cell types compared to CTX-Cy5. However, the CTXA8/CTX uptake ratios revealed that CTXA8 was taken up 2.4–3.5 times more intensively by tumor cells, but only 1.9 times more intensively by normal cells than CTX. This clearly indicated that fluorophore-labeled CTXA8 not only stained tumor cells more intensively than labeled CTX but was also more selective for tumor cells compared to normal cells (Figure 3).

### Cy5-labeled CTXA8 internalizes into U251-MG cells and shows prominent lysosomal colocalization

**FIGURE 4:**
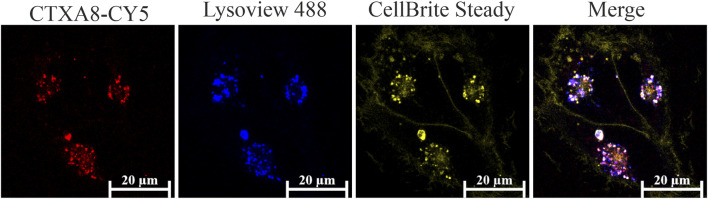
Cellular localization of CTXA8-Cy5 in U251-MG cells by confocal microscopy. U251-MG cells were treated with 0.5 µM CTXA8-Cy5 (red) for 90 min, while lysosomes were counterstained with Lysoview 488 dye (Blue) and plasma membrane was counterstained with membrane impermeable CellBrite Steady dye (yellow). Live-cell image of U251-MG cells revealed the intracellular accumulation of CTXA8-Cy5, and the prominent colocalization of CTXA8-Cy5 with internalized cell surface-derived vesicles, predominantly lysosomes.

Wiranowska et al. demonstrated that the native CTX is mostly internalized by clathrin mediated uptake into glioma cells, and it localizes in the perinuclear Golgi region (Wiranowska et al., 2011). To investigate the internalization route of CTXA8, we assessed the subcellular localization of Cy5-labeled CTXA8 in U251-MG cells by confocal microscopy. As shown in Figure 4, CTXA8-Cy5 predominantly accumulates in lysosomes. Counterstaining of the plasma membrane with CellBrite Steady dye revealed that lysosomes containing CTXA8-Cy5 also colocalize with plasma membrane-derived structures, indicating uptake via plasma membrane–derived vesicles.

### Design and functional characterization of CTX-based CAR constructs incorporating an optimized MMP-2-targeting miniprotein

**FIGURE 5:**
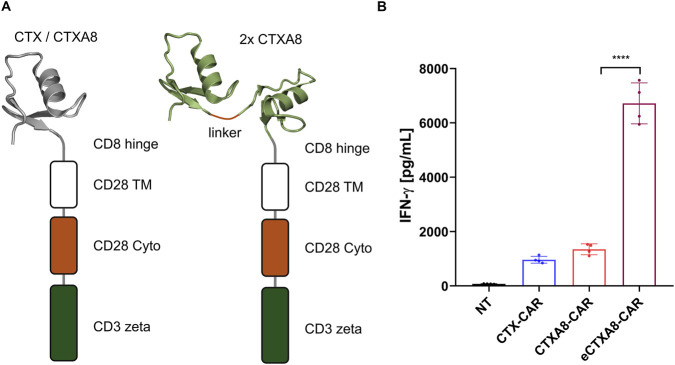
Functional assessment of CTX-based CAR T-cells through IFN-γ secretion. **(A)** Schematic representation of the CAR constructs incorporating wild-type CTX, CTXA8, or tandem CTXA8 units (eCTXA8-CAR). See also Supplementary Figure S2 for additional construct details. **(B)** Functional activation of CAR T-cells measured by IFN-γ secretion following 24-h stimulation with surface-immobilized MMP-2. The data are presented as mean ± SD of N = 4 experiments comparing the IFN-γ secretion of the four different effector cell types. ****p < 0.0001.

To evaluate the therapeutic potential of CTXA8, we hypothesized that it could function as an effective targeting domain within a chimeric antigen receptor (CAR) construct. To test this, we engineered several CAR variants incorporating either wild-type CTX, a single CTXA8 unit, or a tandem arrangement of two CTXA8 units (designated eCTXA8-CAR) (Figure 5A; Supplementary Figure S1).

CAR constructs were assembled using standard molecular cloning techniques. Retroviral particles carrying each CAR variant were generated and pseudotyped, then used to transduce primary human T-cells. Transduction efficiency consistently exceeded 75%, as determined by flow cytometric analysis using an APC-conjugated anti-human CD19 antibody (Supplementary Figure S2). Functional assessment of the CAR T-cells via IFN-γ secretion assays revealed that all three constructs were active, with the eCTXA8-CAR T-cells producing the highest levels of cytokine release, indicating enhanced activation and functionality (Figure 5B).

To directly confirm expression of the engineered CAR constructs, protein lysates from non-transduced (NT), CTX-CAR, CTXA8-CAR, dCTX-CAR, and dCTXA8-CAR T cells were analyzed by Western blot using an anti-CD3ζ antibody (Supplementary Figure S3). In addition to the endogenous CD3ζ band detected at approximately 20 kDa in all T-cell samples, a distinct CAR-associated CD3ζ fusion protein was observed at approximately 60 kDa in all CAR-transduced cells but was absent from NT controls. Higher-molecular-weight species (∼120 kDa), consistent with dimeric or multimeric CAR forms, were also detected and were particularly prominent in the tandem constructs. These findings confirm successful expression of all CAR variants and demonstrate the presence of the expected CAR-associated CD3ζ fusion proteins in engineered T cells.

### Tandem CTXA8-based CAR T-cells exhibit superior cytotoxicity across glioblastoma models

To comprehensively evaluate the tumor-killing efficacy of our engineered CAR T-cell constructs, we performed a series of cytotoxicity assays using both established GB cell lines and primary patient-derived tumor cells. These experiments assessed the functional potency of non-transduced (NT) T-cells, wild-type CTX-CAR T-cells, and eCTXA8-CAR T-cells across a broad range of effector-to-target (E:T) ratios (from 0.003 to 10), with co-culture durations fixed at 24 h.

**FIGURE 6:**
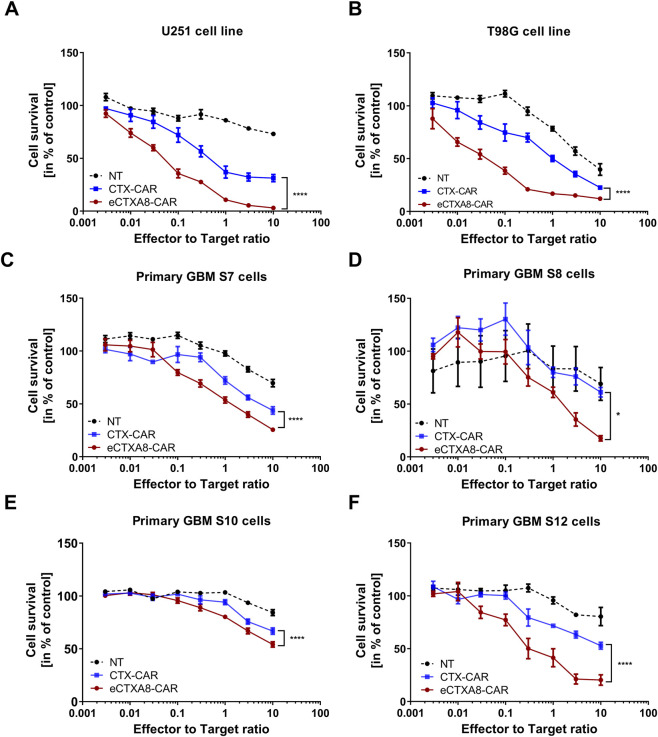
eCTXA8-CAR T-cells exhibit potent cytotoxic activity against immortalized and primary glioblastoma cells. **(A,B)** Cytotoxicity of CAR T-cells against immortalized glioblastoma cell lines U251-MG (A) and T98G (B). **(C–F)** Cytotoxicity of CAR T-cells against patient-derived glioblastoma tumor cells from four individuals: S7 **(C)** S8 **(D)** S10 **(E)** and S12 **(F)** Target glioblastoma cells were co-cultured for 24 h with effector T-cells transduced with either eCTXA8-CAR or wild-type CTX-CAR, or with non-transduced (NT) T-cells, across a range of effector-to-target (E:T) ratios (0.003–10:1). Following co-culture, cell viability was assessed by flow cytometry, identifying live tumor cells as CD45^−^ events. Results are expressed as the percentage of viable target cells relative to untreated controls. Each condition represents the mean ± standard deviation (SD) from two independent CAR T-cell donors (N = 2; independent experiments), with all (E)T ratios tested in technical triplicates. Statistical significance was determined by two-way ANOVA with Tukey’s multiple comparisons test. ****p < 0.0001.

In initial experiments using the U251-MG glioblastoma cell line (Figure 6A), eCTXA8-CAR T-cells exhibited robust and highly efficient cytolytic activity. A marked reduction in tumor cell viability was observed at very low E:T ratios, with 50% viability loss achieved between E:T 0.03 and 0.1. By contrast, CTX-CAR T-cells required a 10-fold higher E:T ratio (≥1) to elicit a comparable reduction in cell survival. Non-transduced T-cells had negligible impact at lower E:T ratios, and only a modest (∼15–20%) reduction in cell viability was detected at the highest E:T ratios tested (Dardevet et al., 2015; McGonigle et al., 2019), indicating minimal nonspecific cytotoxicity from unmodified T-cells.

A parallel experiment using the T98G glioblastoma cell line yielded comparable results (Figure 6B). Again, eCTXA8-CAR T-cells induced potent cytotoxicity, reducing target cell survival below 50% at E:T ratios as low as 0.03–0.1. CTX-CAR T-cells required E:T ratios of ∼1 or higher to achieve similar efficacy, representing approximately a 30-fold difference in functional potency compared to the eCTXA8-CAR construct. Interestingly, NT T-cells showed a slightly higher baseline activity on T98G cells than on U251-MG, but their killing potency remained more than 100-fold lower than that of eCTXA8-CAR T-cells and approximately 5-fold lower than CTX-CAR T-cells. These results confirmed that the enhanced receptor design of eCTXA8-CAR significantly improves tumor cell killing, especially under limiting effector conditions.

Encouraged by the strong performance of eCTXA8-CAR T-cells in immortalized cell lines, we extended our analysis to four patient-derived primary glioblastoma samples (S7, S8, S10, and S12) to assess therapeutic relevance in a more clinically representative setting (Figures 6C–F). While primary GBM cells were generally less sensitive to CAR T-cell-mediated killing than immortalized lines, likely due to intrinsic heterogeneity and resistance mechanisms, eCTXA8-CAR T-cells retained superior activity across all patient samples.

The degree of responsiveness varied between patient samples. S12 cells were the most responsive, showing a 50% reduction in viability at an E:T ratio of just 0.3, highlighting the strong functional engagement of eCTXA8-CAR T-cells even under low effector input. In S7 and S8, 50% viability reduction required E:T ratios between one and 3, while S10 cells—displaying the highest resistance—only responded at the upper end of the E:T ratio range (McGonigle et al., 2019). Despite this, eCTXA8-CAR T-cells consistently outperformed CTX-CAR T-cells in all samples. CTX-CAR T-cells achieved a comparable cytotoxic effect (50% viability loss) only in S12, and only at the highest tested E:T ratio of 10. In the other three primary GBM samples, CTX-CAR T-cells had modest or negligible effects, reinforcing the functional advantage conferred by the tandem CTXA8 design.

Notably, S10 cells exhibited general resistance to both CAR T-cell constructs, but the eCTXA8 variant still produced significantly greater tumor cell killing than its wild-type counterpart. This observation supports the notion that multivalent binding in the eCTXA8 configuration may enhance receptor clustering and synapse formation, potentially overcoming partial resistance mechanisms found in some tumor subtypes.

Because MMP-2 is known to exist both as a membrane-associated and soluble extracellular protein, we investigated whether soluble MMP-2 could interfere with CAR-T-cell activity by acting as an antigen sink. CTX-CAR and eCTXA8-CAR T cells were therefore evaluated against U251-MG and T98G glioblastoma cells at an effector-to-target (E) ratio of 0.1:1 in the presence or absence of 1 μg/mL recombinant human MMP-2 (Supplementary Figure S4). In U251-MG cells, the addition of soluble MMP-2 did not significantly alter the cytotoxic activity of either CTX-CAR or eCTXA8-CAR T cells. Similarly, CTX-CAR T-cell activity against T98G cells was not affected by soluble MMP-2. In contrast, eCTXA8-CAR T cells exhibited significantly enhanced cytotoxicity against T98G cells in the presence of recombinant MMP-2. These findings indicate that soluble MMP-2 does not function as an inhibitory antigen sink under the tested conditions and suggest that eCTXA8-CAR T-cell activity is maintained, and may even be enhanced, in the presence of extracellular MMP-2. Although the mechanism remains to be elucidated, this effect may be related to the increased affinity of CTXA8 for MMP-2, whereby soluble MMP-2 could promote low-level receptor engagement and baseline CAR activation without impairing recognition of cell-associated MMP-2.

In summary, eCTXA8-CAR T-cells demonstrated consistently superior cytotoxic activity compared with CTX-CAR T-cells and NT controls across all tested GBM models. Their enhanced potency at low E:T ratios and sustained activity in primary patient-derived tumors highlight their therapeutic potential and warrant further preclinical and translational evaluation. These findings also support the broader concept that rationally engineered miniprotein-based CARs can address key limitations of conventional antibody-derived CAR constructs, particularly in the context of solid tumors such as glioblastoma.

### Reduced expression of pro-apoptotic markers and upregulated MMP-2 expression in patient-derived primary glioblastoma cells

**FIGURE 7:**
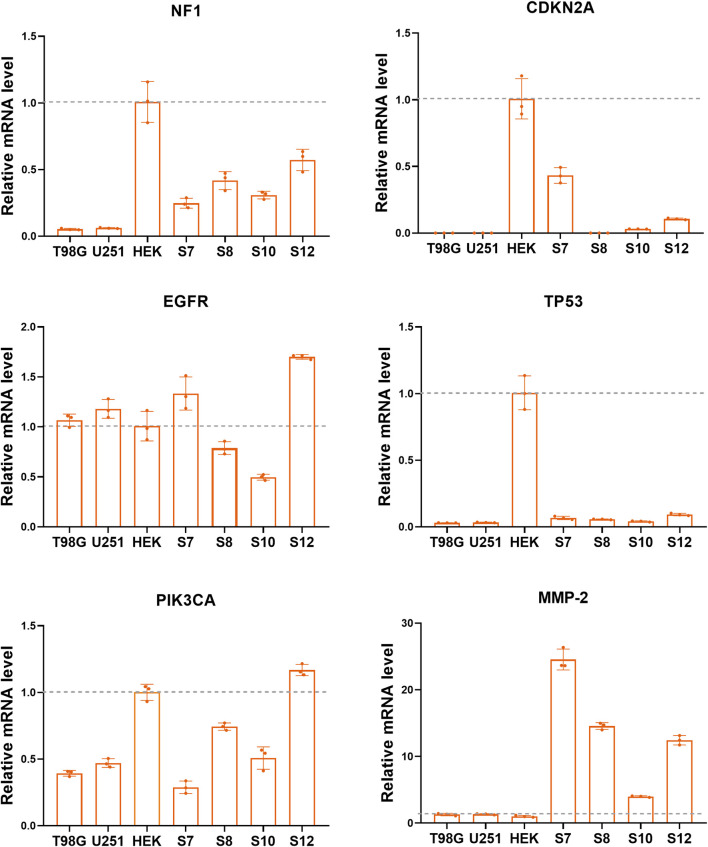
Gene expression analysis of the tested target cell lines and patient-derived primary GBM cells. Expression of key genes involved in tumor suppression and apoptosis regulation was analyzed, including cellular tumor antigen p53 (*TP53*), cyclin-dependent kinase inhibitor 2A (*CDKN2A*), and phosphatidylinositol-4,5-bisphosphate 3-kinase catalytic subunit α (*PIK3CA*). Additionally, the expression of epidermal growth factor receptor (*EGFR*), neurofibromin-1 (*NF1*), and matrix metalloproteinase-2 (*MMP-2*) was examined. Data are presented as means ± SD with superimposed scatter plots.

In accordance with the 2021 WHO classification of glioblastomas based on molecular features (Park et al., 2023), the expression of key genes involved in tumor suppression and apoptosis regulation, specifically *TP53, CDKN2A, PIK3CA*, was analyzed. In addition, the expression of other target genes, including *EGFR*, *NF1* and *MMP-2*, was also investigated (Figure 7, Gene expression data in the Supplementary Material, and 10.5281/zenodo.17542252).

Gene expression analysis showed that NF1 mRNA levels were more than twofold lower in GBM-derived immortalized cell lines and primary GBM cells compared to HEK-293 cells. CDKN2A expression was absent in U251-MG, T98G, and S8 cells, consistent with reported homozygous deletions in U251 clones. TP53 expression was nearly undetectable across all GBM cells and immortalized cells lines relative to HEK293T cells. PIK3CA expression was reduced to ∼50% of HEK293T levels in U251-MG and T98G cells, while patient-derived GBM cells displayed variable expression. MMP-2 levels were elevated in patient-derived GBM cells, with S10 identified as a low MMP-2 population and S7, S8, and S12 as high MMP-2 populations. Immunolabeling confirmed astrocytic tumor origin: GFAP staining was positive in >90% of cells from three subjects, while FAPα and CD90 expression varied between donors (data not shown). The heterogeneous expression profiles indicated preservation of tumor heterogeneity in the primary GBM-derived cell populations.

## Discussion

Miniproteins, owing to their unique structural and functional properties, represent promising therapeutic modalities for targeting biological structures that remain challenging or inaccessible to conventional treatments. One such target is matrix metalloproteinase-2 (MMP-2), an enzyme implicated in glioblastoma (GB) progression. Attempts to selectively inhibit MMP-2 with small molecules have largely failed due to the high sequence similarity of its catalytic domain with other members of the MMP family. Chlorotoxin (CTX), a cysteine-rich miniprotein originally derived from scorpion venom, has emerged as a candidate for selective MMP-2 binding and broader GB targeting. In our previous study, we demonstrated that CTX binds to MMP-2 with affinity in the high-nanomolar range (Farkas et al., 2023). Importantly, this interaction does not inhibit MMP-2’s enzymatic function, indicating that CTX binds outside the catalytic site. Othman et al. (2017) predicted that CTX binds near the fibronectin-like domains of MMP-2, rather than the catalytic site. This interaction is unusual for miniproteins, which typically target small clefts, active sites, or ion channel pores with very high affinity. Although CTX evolved to immobilize arthropod prey, its incidental affinity for human proteins such as MMP-2 may be exploited for therapeutic applications.

In addition to MMP-2, we previously showed that CTX binds to neuropilin-1 (NRP1) and the CLC3 chloride channel (Farkas et al., 2023), raising concerns about specificity. To improve the affinity and selectivity of CTX toward MMP-2, we generated a phage display library by recombining homologous segments from related miniproteins within the same scaffold family, instead of using conventional random mutagenesis. The resulting library contained a theoretical diversity of 1.3 million variants, confirmed by next-generation sequencing, and was screened against immobilized MMP-2 through iterative biopanning cycles.

From this screen, we identified CTXA8, which displayed significantly improved MMP-2 binding. Recombinant Cy5-labeled CTXA8 exhibited a 4.4-fold increase in affinity (IC_50_: 160 nM) compared to CTX. Binding specificity assays showed that CTXA8 did not bind NRP1, CLC3, or other tested proteins, confirming its improved selectivity. However, residual detectable binding to CLC3 may reflect partial preservation of structural motifs shared between CTX and CTXA8 despite substantial optimization toward MMP-2 selectivity.

Cell-based studies further validated these findings. Cy5-labeled CTXA8 produced more intense staining in multiple MMP-2-positive cancer cell lines–including U251-MG (glioblastoma), Panc-1 (pancreatic cancer), A375 (melanoma), and Sk-Br (breast cancer) – compared to CTX. Importantly, uptake in non-cancerous HDFa cells was minimal. Quantitative analysis revealed 2–4-fold higher uptake of CTXA8-Cy5 versus CTX-Cy5 across all cancer cell lines, supporting the conclusion that enhanced cellular binding was driven by improved MMP-2 recognition. However, a limitation of the present study is that membrane-associated MMP-2 was not directly quantified by flow cytometry or immunofluorescence. Because MMP-2 surface localization depends on complex formation with MT1-MMP and TIMP2 as well as post-transcriptional trafficking mechanisms, mRNA abundance alone may not fully reflect surface-accessible antigen density.

Confocal microscopy further demonstrated lysosomal colocalization of CTXA8, consistent with prior evidence that MMP-2 cycles through extracellular localization, rendering it accessible to CAR T-cells. Plasma membrane staining with a membrane-impermeable dye confirmed that lysosomes containing CTXA8 originated at the cell surface. Although the complete endosomal trafficking pathway was not explored, these findings indicate that CTXA8 follows a similar internalization route as native CTX.

Beyond diagnostic and imaging applications—such as the CTX derivative tozuleristide, currently developed for intraoperative fluorescence-guided surgery (Yamada et al., 2021)—CTXA8 offers potential as a targeting domain in cellular immunotherapy. In particular, CAR T-cell therapy directed against MMP-2-positive GB has gained traction. A CTX-derived CAR (CLTX-CAR) was recently shown to mediate tumor-specific recognition and cytotoxicity while minimizing off-tumor toxicity, with preclinical models demonstrating improved survival in GBM-bearing mice (Wang D. et al., 2020).

Building on this concept, we engineered a CAR construct incorporating CTXA8 as the binding domain. The construct used a CD8 hinge and CD28 costimulatory domain, as described by Wang D. et al. (2020). To enhance functionality, we introduced a novel modification by duplicating the extracellular CTXA8 domain, drawing inspiration from a patent (Chaudhary et al., 2018) describing enhanced antitumor effects from CARs with multiple ectodomains. This design was intended to increase avidity and potentially engage alternative activation mechanisms.

Compared with conventional scFv-based CARs, cystine-dense miniproteins offer several potential advantages, including compact size, enhanced structural stability, reduced aggregation tendency, and resistance to proteolytic degradation. Unlike many scFvs, which contain hydrophobic cores that may contribute to misfolding and tonic CAR signaling, miniproteins are stabilized primarily by disulfide-bonded cystine-knot architectures. These properties may support more stable CAR surface expression and potentially reduce ligand-independent activation.

CAR expression was initially assessed by flow cytometric detection of the co-expressed truncated CD19 marker, which demonstrated efficient transduction of all CAR constructs. To further validate expression at the protein level, Western blot analysis was performed using an anti-CD3ζ antibody. This analysis confirmed the presence of CAR-associated CD3ζ fusion proteins of the expected molecular weight in all engineered T-cell populations. In addition to the endogenous CD3ζ band, higher-molecular-weight species corresponding to CAR-associated proteins were detected, with prominent multimeric forms observed under non-reducing conditions. Together, the CD19 flow cytometry and Western blot data provide complementary evidence for successful expression of both single and tandem CTX-derived CAR constructs.

Our engineered eCTXA8-CAR T-cells exhibited potent antitumor activity. In co-culture assays with U251-MG, T98G, and primary patient-derived GBM cells, eCTXA8-CAR T cells induced >90% tumor cell death within 24 h at an effector-to-target (E:T) ratio of 1:1. By comparison, GD2-targeted CAR T-cells—a competing candidate for GB therapy—achieved only 70%–75% cytotoxicity at a higher E:T ratio of 5:1 (Chiavelli et al., 2024).

eCTXA8-CAR T-cells showed strong antitumor activity across immortalized and patient-derived GB models despite variability in NF1, CDKN2A, TP53, PIK3CA, and stem-like markers. The variable responses observed in patient-derived GBM cells likely reflect differences in MMP-2 expression, underscoring a central challenge in solid-tumor CAR T-therapy (Marofi et al., 2021). It is essential to recognize that MMP-2 differs fundamentally from classical CAR targets, as it is not a constitutively expressed membrane protein. Instead, its presence on the cell surface depends on the formation of complexes with membrane-type MMPs (particularly MT1-MMP) and regulatory proteins such as TIMP2, which mediate pro-MMP-2 activation and localization (Stetler-Stevenson, 2008). Consequently, mRNA expression levels do not necessarily predict the amount of membrane-associated MMP-2, as posttranscriptional and adapter-mediated mechanisms—including intracellular trafficking and recycling through clathrin- and PDZ-dependent pathways—can substantially influence surface availability (Hey et al., 2022). Furthermore, epigenetic control of MMP-2 and MT1-MMP promoters, together with variations in adapter protein expression, likely contributes to the heterogeneous MMP-2 presentation observed across primary GBM cultures (Chernov et al., 2009). These factors, taken together, may underline the observed discrepancies between transcriptional data, MMP-2 surface expression, and CAR-T cytotoxic responses in our study. The results of the gene expression analysis are particularly significant given the current limitations of CAR T-therapy in GB, including tumor heterogeneity, antigen escape, and an immunosuppressive microenvironment (Montoya et al., 2024; Salinas et al., 2020). The robust efficacy of eCTXA8-CAR T-cells across diverse GBM samples suggests that MMP-2-targeted CARs may help overcome these barriers by improving tumor specificity and accommodating inter-patient variability in antigen expression. Although the precise interaction interface of eCTXA8 on MMP-2 remains to be experimentally defined, previous computational studies on native CTX suggest binding near the fibronectin-like domain rather than the catalytic pocket. Since CTXA8 was derived from CTX through directed evolution while retaining the same cystine-knot scaffold, it is plausible that a related interaction surface is preserved. Nevertheless, detailed structural characterization using alanine scanning, mutagenesis, or computational docking will be important in future studies.

An important consideration for MMP-2-targeted CAR-T therapies is the presence of soluble MMP-2 within the tumor microenvironment, which could theoretically act as an antigen sink and impair tumor recognition. To address this possibility, we evaluated CAR-T-cell activity in the presence of recombinant soluble MMP-2. Unexpectedly, soluble MMP-2 did not reduce the cytotoxic activity of either CTX-CAR or eCTXA8-CAR T cells. Moreover, enhanced cytotoxicity of eCTXA8-CAR T cells was observed against T98G cells in the presence of soluble MMP-2. Although the underlying mechanism remains unclear, one possible explanation is that the increased affinity of eCTXA8 enables productive interaction with soluble MMP-2, resulting in low-level receptor engagement and priming of CAR-T-cell activity without compromising recognition of membrane-associated MMP-2. These findings suggest that soluble MMP-2 may not represent a major obstacle for CTXA8-based CAR approaches and support further investigation of CAR activation dynamics in physiologically relevant glioblastoma models.

In summary, our study highlights the feasibility of engineering target-specific miniproteins through recombination-based library design and high-throughput screening. This platform enables improved affinity and selectivity while remaining adaptable to a wide range of therapeutic targets. CTXA8 exemplifies the clinical potential of this approach: it combines enhanced MMP-2 specificity with translational applications, from imaging to CAR T-cell therapy. Incorporating CTXA8 into a multivalent CAR design resulted in highly effective antitumor activity in GBM models, providing a strong rationale for further preclinical development and potential clinical translation.

## Data Availability

The data presented in the study are deposited in the Zenodo repository, accession number: 10.5281/zenodo.17542252.
