# Supplementary material for: Directed evolution of chlorotoxin enhances MMP-2 recognition and improves CAR-T-cell activity in glioblastoma models in vitro
Source: Front Bioeng Biotechnol. 2026 Jul 20;14:1866928. doi: 10.3389/fbioe.2026.1866928 (PMC13429609; doi:10.3389/fbioe.2026.1866928)
Supplement: Supplementary file 1 [file Supplementaryfile1.docx]

Supplementary Material

| **Supplementary Table 1. The list of miniprotein homologues for building the combinatorial phage library.** A total of 28 homologous miniproteins from the same scaffold family were chosen for a combinatorial phage display library. The UniProt entries are listed, with chlorotoxin (CTX) at the top. Based on homology and feasibility, the amino acid sequences were divided into three segments (A, B, and C), linked by nucleotide sequences corresponding to cysteine residues 3^rd^ and 6^th^. To increase diversity, each segment was split into two subsegments, resulting in 121 unique segment A, 94 unique segment B, and 121 unique segment C, giving a total of 1,376,254 variants. |
| --- |

| **Supplementary Figure 1. Amino acid sequence of the eCTXA-CAR construct.** The expression construct is composed of a signal sequence (highlighted in green), two tandem CTXA8 targeting miniproteins (in light blue) connected by linker-218 (in magenta), followed by a CD8 hinge region (in red), a transmembrane spanning helix structure, denoted as CD28tm (in dark cyan), the cytoplasmic part of CD28 (marked as CD28cyto in deep purple), then CD3zeta (in dark red) connected to a truncated CD19 (CD19t, in dark green) via the self-cleavage sequence T2A (in dark blue). The red asterisk marks the stop codon (TGA). The miniprotein coding sequence was ordered as a synthetic construct from Eurofins Genomics cloned into pEX-A128 cloning vector, then inserted into a NcoI-KasI linearized pSFG retroviral vector harboring the CAR construct. |
| --- |
| Signal sequence 1st CTXA8  MLLLVTSLLLCELPHPAFLLIPAMCMPCSSSDHESARRCRDCCGGYGKCFGYQCLCNRRRGS  Linker-218 2nd CTXA8  TSGSGKPGSGEGSTKGAMCMPCSSSDHESARRCRDCCGGYGKCFGYQCLCNRRRTTTPAPRP  CD8 hinge CD28tm  PTPAPTIASQPLSLRPEACRPAAGGAVHTRGLDFACDMFWVLVVVGGVLACYSLLVTVAFII  CD28cyto  FWVRSKRSRGGHSDYMNMTPRRPGPTRKHYQPYAPPRDFAAYRSGGGRVKFSRSADAPAYQQ  CD3zeta  GQNQLYNELNLGRREEYDVLDKRRGRDPEMGGKPQRRKNPQEGLYNELQKDKMAEAYSEIGM  T2A  KGERRRGKGHDGLYQGLSTATKDTYDALHMQALPPRASRAEGRGSLLTCGDVEENPGPMPPP  CD19t  RLLFFLLFLTPMEVRPEEPLVVKVEEGDNAVLQCLKGTSDGPTQQLTWSRESPLKPFLKLSL  GLPGLGIHMRPLAIWLFIFNVSQQMGGFYLCQPGPPSEKAWQPGWTVNVEGSGELFRWNVSD  LGGLGCGLKNRSSEGPSSPSGKLMSPKLYVWAKDRPEIWEGEPPCLPPRDSLNQSLSQDLTM  APGSTLWLSCGVPPDSVSRGPLSWTHVHPKGPKSLLSLELKDDRPARDMWVMETGLLLPRAT  AQDAGKYYCHRGNLTMSFHLEITARPVLWHWLLRTGGWKVSAVTLAYLIFCLCSLVGILHLQ  RALVLRRKRKRMTDPTRRF* |

| **Supplementary Figure 2. Transduction efficacy measurement of CAR constructs.** |
| --- |
| **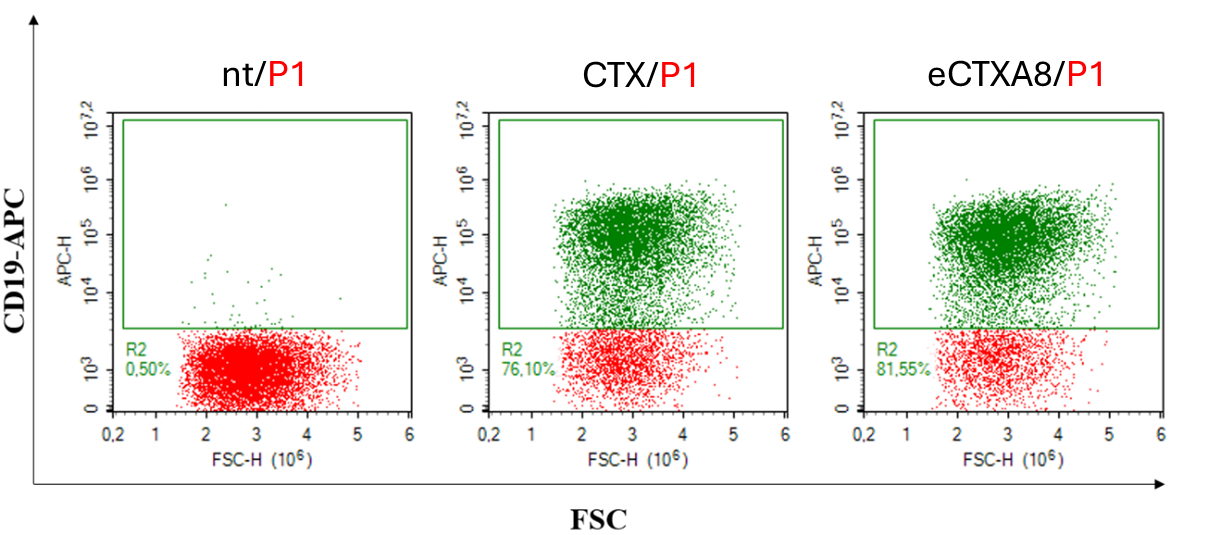** |

| **Supplementary Table 2. Primer design for primary GMB cell characterization.** For each gene, the sequence of forward and reverse primers is listed, the nucleotide sequences read from 5’ to 3’. | | | |
| --- | --- | --- | --- |
|  |  |  |  |
| **Target Genes** | **Primer sequences (forward, reverse)** |  |  |
| *NF1* | 5’-CAACAACTTCAATGCAGTCTTTAGTCGC  5’-GCTAACTGCGCAACCTTCTTTAGG |  |  |
| *CDKN2A* | 5’-ATCGCGATGTCGCACGGTAC  5’-CCTGTAGGACCTTCGGTGACTGATG |  |  |
| *EGFR* | 5’-GACTGAAGGAGCTGCCCATGAG  5’-CCATTGGGACAGCTTGGATCACAC |  |  |
| *PIK3CA* | 5’-GAGTACCTTGTTCCAATCCCAGGTGG  5’-GTTTATATTTCCCCATGCCAATGGACAG |  |  |
| *TP53* | 5’-GCAGTCAGATCCTAGCGTCGAG  5’-CTGGACCTGGGTCTTCAGTGAACC |  |  |
| *MMP-2* | 5’-CCTGACCAAGGGTACAGCCTGTTCC  5’-GGTGCCAAGGTCAATGTCAGGAGAGG |  |  |
| *ACTB* | 5’-CAGGATGGAGGGGAAGACGG  5’-CGCGCCAGCTCACCGACGG |  |  |
| **Supplementary Figure 3. Western blot confirmation of CAR-specific and endogenous CD3ζ expression in engineered T cells.** Protein lysates from non-transduced (NT) T cells and T cells expressing CTX-CAR, CTXA8-CAR, eCTX-CAR, or eCTXA8-CAR constructs were analyzed by western blot using an anti-CD3ζ antibody. Endogenous CD3ζ was detected at approximately 20 kDa in all samples. CAR-associated CD3ζ fusion proteins were detected at approximately 60 kDa, corresponding to the expected molecular weight of the CAR constructs. Higher-molecular-weight bands (~120 kDa) likely represent dimeric or multimeric forms of the CAR-associated CD3ζ fusion proteins. Increased intensity of the higher-molecular-weight species was observed in tandem CAR constructs (dCTX-CAR and dCTXA8-CAR), consistent with their larger extracellular domains. Molecular weights are indicated on the right. | | | |
| **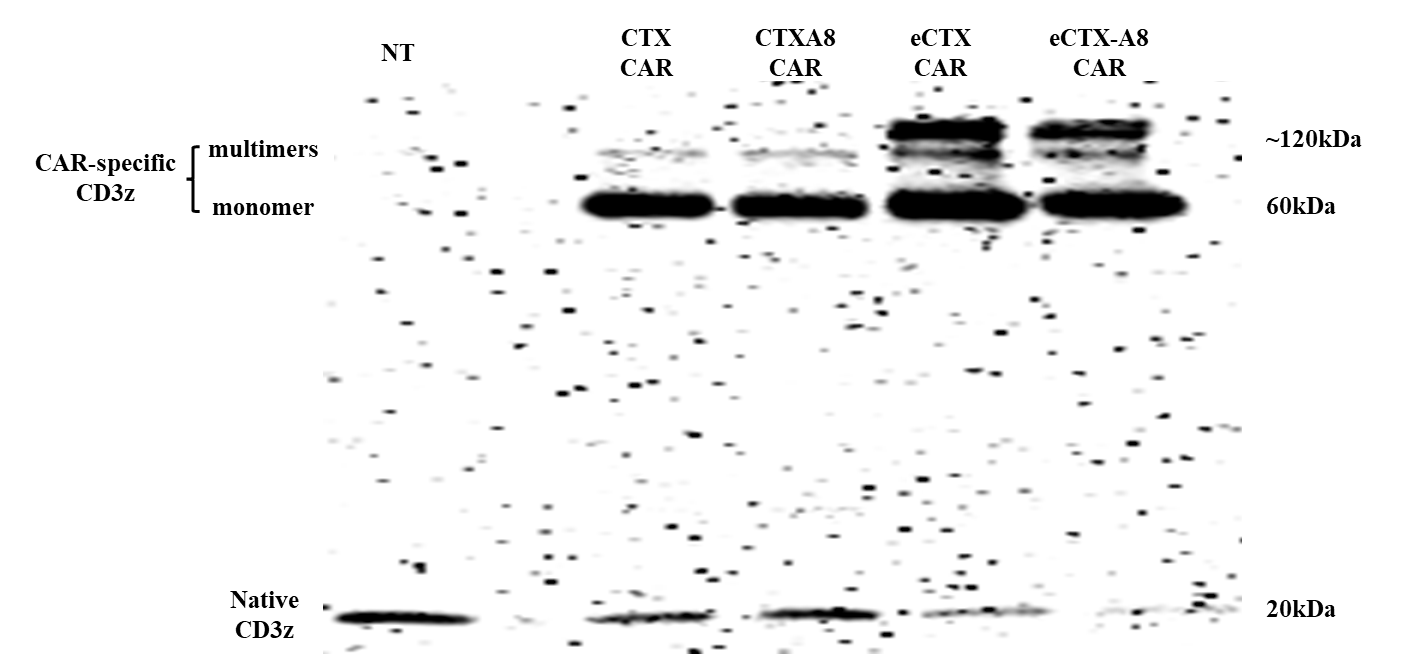** | | | |

| **Supplementary Figure 4. Soluble MMP-2 does not impair and may enhance eCTXA8-CAR T-cell cytotoxicity against glioblastoma cells.** Left panel: Cytotoxic activity of CTX-CAR and eCTXA8-CAR T cells against U251 glioblastoma cells at an effector-to-target (E:T) ratio of 0.1:1 in the absence or presence of 1 μg/mL recombinant human MMP-2. The addition of soluble MMP-2 did not significantly alter the cytotoxic activity of either CAR construct. Right panel: Cytotoxic activity of CTX-CAR and eCTXA8-CAR T cells against T98G glioblastoma cells under identical conditions. While soluble MMP-2 had little effect on CTX-CAR T-cell activity, the presence of recombinant MMP-2 enhanced the cytotoxic activity of eCTXA8-CAR T cells. Data are presented as mean ± SD from independent experiments. Statistical analysis was performed using a two-tailed Student’s t-test. ns, not significant; *p < 0.05. |
| --- |
| **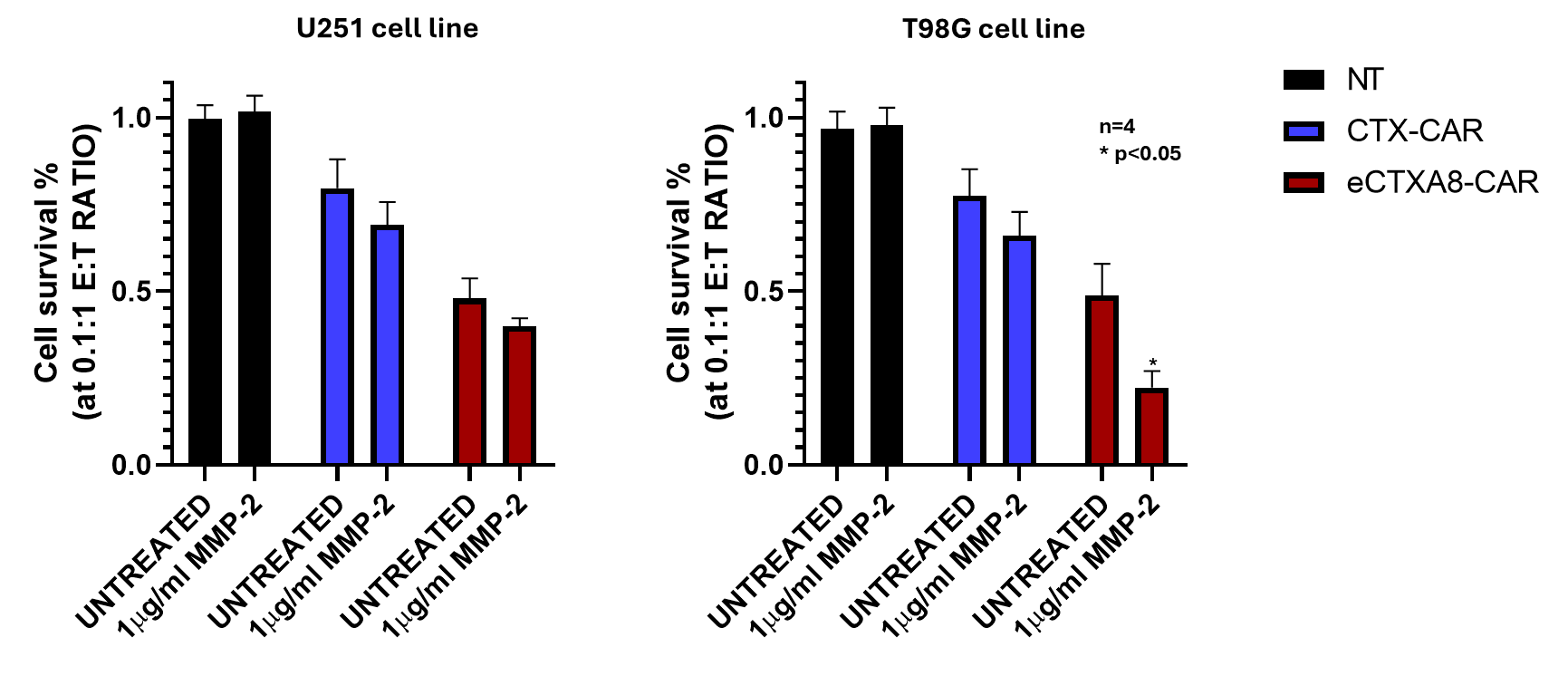** |
